# Dialogic reading at age 2 is linked to frontal activation related to executive function at age 5: An fNIRS study

**DOI:** 10.1371/journal.pone.0351177

**Published:** 2026-06-15

**Authors:** Ming Yean Sia, Chia-Feng Lu, Ovid J. L. Tzeng, Shinmin Wang

**Affiliations:** 1 Department of Child and Family Science, National Taiwan Normal University, Taipei, Taiwan; 2 Department of Biomedical Imaging and Radiological Sciences, National Yang Ming Chiao Tung University, Taipei, Taiwan; 3 Linguistic Institute, Academia Sinica, Taipei, Taiwan; 4 College of Humanities and Social Sciences, Taipei Medical University, Taipei, Taiwan; University of Calgary, CANADA

## Abstract

This study investigates the relationship between children’s dialogic reading (DR) experiences with parents at age 2 and their frontal neural responses related to executive function (EF) at age 5. To assess how the intensity of DR influences brain development, we quantitatively measured parental engagement in DR when children are at 2 years of age. Neural activations in frontal regions associated with EF were evaluated using functional near-infrared spectroscopy when children reached age 5. Our results reveal a significant positive correlation between parental dialogic interaction during shared book reading at age 2 and the activation of key brain regions related to EF – the bilateral dorsolateral prefrontal cortex and the bilateral inferior frontal gyrus – during a Dimensional Change Card Sort (DCCS) task at age 5. This correlation persisted even after controlling for maternal education and children’s expressive vocabulary, indicating a robust relationship between early DR experiences and subsequent neural correlates of EF. The results suggest that early DR may help cultivate the neural infrastructure necessary for EF development. By focusing on DR at a young age and assessing neural activity during a classic EF task, the DCCS, our findings contribute additional evidence regarding the role of DR in shaping neural development associated with EF. These results highlight the importance of encouraging interactive DR practices in early childhood, as they not only support language development but also strengthen the neural pathways crucial for cognitive skills essential for academic success.

## Introduction

As children’s first learning environment is typically their home, parent-child interaction is important in facilitating the development of children. One common activity that promotes parent-child interaction is shared book reading. There is considerable behavioural evidence demonstrating that early interactive shared reading, also known as dialogic reading (DR), enhances children’s linguistic abilities later in life [1]. In addition, emerging evidence [2] indicates that children’s shared reading or DR experiences are related to changes in neural activity during language processing tasks [3–7], attention tasks [8] and sensory prediction tasks [9], highlighting the neural benefits of these practices for young children. Despite this progress, much remains unknown about the role of DR in neural development. One understudied area is how DR experience might contribute to children’s neural development associated with higher cognitive functions, such as executive function (EF). EF is the cognitive ability that allows one to control and adapt one’s behaviour in response to the environment and task at hand, which is important to many aspects of life, such as school readiness, school success, job success, marital harmony and public safety [10]. To elucidate this issue, the present study explored the longitudinal association between children’s DR experience at age 2 and their neural responses related to executive function at age 5.

As the name suggests, DR stresses on children’s active rather than passive participation in reading, where parents are encouraged to have a dialogue with their child about the book that they are reading [11]. To stimulate DR, parents are usually taught the acronym PEER [12], which stands for *prompt* (inviting children to express themselves about the book’s content), *evaluate* (praising or correcting children’s response), *expand* (providing more details to children’s response), and *repeat* (reinforcing children’s learning by asking them to repeat the correct answer). The strategy prompt can be achieved through yet another acronym known as CROWD, i.e., *completion* (leaving a sentence hanging for children to complete), *recall* (asking children about an information that was previously read), *open-ended* (asking children questions that encourage them to respond to the book in their own way), *WH questions* (prompting children using WH question words) and *distancing* (asking children to relate the book’s content with their own experience). Simply put, DR provides children with opportunities to experience “serve-and-return” interactions with parents.

Thus, during DR with their parents, children would actively engage not only their language skills and basic attentional networks (e.g., visual and auditory attention) but also various higher cognitive functions, particularly EF. These include working memory [i.e., temporarily storing and manipulating information; 13, 14], inhibitory control [i.e., inhibiting irrelevant behaviour or prepotent responses; 15] and mental shifting [i.e., switching between tasks or rules; 16]. For example, to maintain the serve-and-return interaction, children need to temporarily remember information from the book that is relevant to the unfolding dialogue, demonstrating working memory. When prompted with questions linking book contents to their own personal experience, children must shift back and forth between the fictional world in the book and their real-world experiences, exemplifying mental shifting. Moreover, children need to hold back their impulses to speak out of turn, waiting their chance to respond and pausing when it is their parents’ turn to talk, which highlights the use of inhibition.

These EF components undergo substantial development during the early years [10]. The ability to hold and manipulate information in working memory typically emerges by age 3 [17] and continues to develop progressively from age 4 through age 14 [18]. Children aged 3–4 years often struggle to inhibit prepotent responses unless are forced to delay their responses [19], whereas by age 7, their inhibitory control approaches adult-like levels [20]. The ability to flexibly switch between tasks or mental sets typically emerges around age 5 [21] and is the last major EF component to develop [10].

In fact, EF has long been found to associate with the prefrontal cortex (PFC), which occupies nearly a third of the cerebral cortex, and is the last brain region to fully mature. Structural MRI research has demonstrated that PFC volume increases steadily until around 8 years old, then expands rapidly between age 8 and age 14, reaching maturity around age 18 [22]. Despite this protracted maturation, early childhood is a period during which PFC begins to undergo notable structural changes. For example, the maximum synaptic density (the peak in synaptogenesis) in the PFC is reached between 12 and 18 months of age, followed by a gradual process of synapse elimination that continues into mid-adolescence [23]. Myelination in the PFC begins around 6–8 months, enabling more efficient and synchronised communication between neural systems [24]. Similarly, glucose metabolism in the PFC, a vital biochemical process through which cells break down glucose to produce energy for cellular functions, begins to increase around 6–8 months and continues to rise rapidly until approximately 3–4 years of age [25]. These early changes in synaptogenesis, synaptic pruning, myelination and metabolism in PFC during early childhood may provide an important foundation for subsequent PFC development, making early childhood a window of opportunity to support the emergence of PFC functions.

Indeed, emerging evidence to date indicates that environmental inputs such as DR can shape prefrontal circuitry during early childhood (e.g., 4, 26). For instance, in a fMRI study, Hutton et al. [4] found that higher levels of verbal interactivity during maternal shared reading are related to greater brain activations not only in the language network (the parietal-temporal-occipital region) but also in areas implicated in EF (the lateral frontal pole and anterior insular) during a passive story listening task in low-SES 4-year-old girls in a cross-sectional study. Similarly, Ohgi et al. [26] used functional near-infrared spectroscopy (fNIRS) and discovered that 3-year-old children showed activations in the bilateral frontal areas during interactive parent-child book reading.

To directly assess EF-related neural benefits induced by DR, Twait et al. [8] conducted a 6-week intervention study in which children were read to by a stranger. They compared the P300 and N200 event-related potential (ERP) amplitudes in the congruent and incongruent conditions of the Attentional Network Task among 4-years-old who received either DR or screen-based story-telling intervention. The P300 component reflects orientation attention (basic-level attention grounded in bottom-up processing), while the N200 component reflects executive-control abilities (high-level attention based on top-down processing). Results showed that children in the DR group had a smaller P300 amplitude difference between congruent and incongruent conditions, suggesting improved orientating attention compared to the screen-based group. The DR group also displayed a smaller N200 difference relative to the screen-based group, though the group difference did not reach statistical significance. These authors argued that a longer intervention period beyond six weeks might be necessary for DR to effectively enhance executive function [8], although how much DR exposure is required to produce lasting neural changes remains unknown.

While prior research significantly contributes to our understanding of the impacts of DR on the improved neural function associated with EF, two key gaps remain. The first pertains to timing. Both Hutton et al. [4] and Twait et al. [8] focused on 4-years-old, leaving a gap in understanding how earlier DR experiences at home might influence the neural correlates of EF changes later in life. Addressing this question is particularly informative, as longitudinal neuroimaging research points to an early window of opportunity (i.e., around 1.5–2 years of age), during which environmental input may exert especially strong and lasting effects on brain organization [27,28]. The current study aims to address this gap by investigating the impact of DR at age 2 on the neural correlates of EF at age 5, while accounting for the influences of maternal education and children’s language skills. These covariates were included because previous research has shown that EF development in young children is closely related to their language skills [29] and that individual differences in brain developmental trajectories are associated with maternal education [27].

We focus on DR at age 2 not only because prior brain research highlights 1.5–2 years of age as a period of heightened sensitivity to environment input in general [27,28], but also because behavioural evidence revealing that shared book reading experience before 2 years can have enduring effects. Specifically, early shared reading predicts children’s vocabulary size at age 3 [30] and broader cognitive competencies at age 5 including rhyming, verbal comprehension and concept formation [29,31]. In addition, gene-environment interaction research revealed that shared book reading as early as 1 year old can serve as a protective factor for children genetically at risk for delayed language development, potentially through modulation of dopaminergic and serotonergic systems [32]. Taken together, these findings suggest the significance of investigating the enduring effects of DR at 2 years. Furthermore, we target five-year-olds for measuring PFC functions because recent fNIRS evidence indicates that the measurable neural benefits of shared or dialogic reading become more pronounced in older children (i.e., after approximately 30 months of age) [6].

The second gap in previous research concerns the limitations of existing neuroimaging methods and study designs. While ERP offers excellent temporal resolution, it lacks the ability to precisely determine the exact origins of brain activities. Hutton et al. [4] used fMRI, which provides spatial information, but in their study, they measured brain activity during story listening rather than an explicit EF task that could directly trigger EF. Conversely, Twait et al. [8] employed an EF task but did not measure the level of interactivity during DR as Hutton et al. did in their study, which could be a crucial factor in understanding the nuances of impacts of DR practices.

To address the second gap, we employed fNIRS, an advanced neuroimaging technique that offers superior spatial resolution compared to electroencephalography (EEG), such that we were able to identify specific brain regions activated during EF tasks, and provide further insights into the neural dynamics affected by DR. Furthermore, we quantified the level of parental interactivities during DR, in order to understand its impact. By integrating these elements, the current study would provide a more comprehensive understanding of how DR experiences correlates with neural responses associated with EF in early childhood.

While many tasks exist to index EF, the current study used the Dimensional Change Card Sort [DCCS; 33], a child-adapted version of the Wisconsin Card Sort Test [34], for two reasons. First, the DCCS taps on EF components possibly involved in DR. In this task, children first sort cards by one dimension (e.g., shape) and then by another (e.g., colour). Successful performance requires the use of working memory to apply the current sorting rule, inhibition to avoid interference from the previous rule, and cognitive flexibility to shift between rules. Thus, DCCS can be considered an appropriate measure of general EF.

Second, DCCS has been successfully used in past research to assess brain functions related to EF in young children [33,35–38], where developmental changes in neural responses linked to DCCS performance are also captured. In particular, Moriguchi and colleagues used fNIRS to investigate the neural correlates of DCCS, and results of their research consistently revealed that preschoolers recruit the dorsolateral prefrontal cortex (DLPFC) during the task [39]. Children between 3 and 5 years of age who successfully sorted cards by a new dimension showed stronger activation in the inferior frontal gyrus (IFG) as they aged [21,40]. These data suggest that greater prefrontal activations indicate improved EF skills during preschool years. The current study focused on the brain activation in the DLPFC and IFG induced by DCCS to index neural correlates of EF in children.

In sum, the current study aimed to examine the link between children’s DR experiences at age 2 and their frontal neural responses during DCCS at age 5. DR experiences were measured at age 2, a possible window of opportunity during which DR may have strong and lasting impacts on prefrontal development [27,28]. The decision to examine brain activation at age 5 was based on evidence that the neural benefits of shared or dialogic reading become more measurable in older children [6] and that children of age 5 begin to display adult-like activation patterns when performing DCCS. Specifically, both 5-year-old children and adults show successful performance and bilateral inferior prefrontal cortex during the DCCS [21]. By examining the longitudinal association between DR at age 2 and EF-related neural activation at age 5, the current study extends prior work that has primarily focused on children aged 3–4 and contributes novel evidence on the developmental time frame during which DR may have its enduring neural effects.

We hypothesised that greater parental involvement in DR would provide children more opportunities to utilise neuronal pathways in frontal regions associated with EF, leading to stronger prefrontal activations later on compared to peers with less DR experience. If our hypothesis holds, we would expect a positive link between children’s DR experiences and the strength of their frontal activations. To quantify DR experience, we video-recorded and coded shared reading sessions between parents and their two-year-olds using the PEER coding scheme designed by Chang et al. [41]. Frontal brain activity during DCCS was measured using fNIRS, with DLPFC (BA9/46) and IFG (BA44/45/47) as the regions of interest because these regions have been previously linked to EF during DCCS [39].

## Materials and methods

### Participants

The present study is part of a longitudinal study in which 67 parents of 6-month-old infants were initially given an age-relevant book to be read with their child. This was done at every six-month interval till the child was 24 months old. The first testing date (i.e., when the children were 6 months old) ranged from 13/12/2017 to 30/12/2019. The data relevant to the present study were the shared reading recordings when the children were 24 months old.

Of these 67 parent-child dyads, 53 parent-child dyads agreed to take part in the DCCS task when the children were around 4–5 years old. The DCCS testing date ranged from 8/4/ 2023–13/5/2023 and was conducted in a quiet room within a school located in the county where the participants lived. Seven out of the 53 children were excluded from the analyses due to the following reasons: refusal to wear the fNIRS caps (*n* = 2), noisy data in most channels (*n* = 2), and failure in the DCCS task (*n* = 3) (see *fNIRS data processing* for detailed exclusion criteria). After these exclusions, 46 children remained. Seven out of the 46 children did not have shared reading data due to technical problems during recordings (i.e., the camera failed to record the reading session). Thus, the final sample consisted of 39 parent-child dyads (19 girls and 20 boys; shared reading age: *M* = 24 months, *SD* = 0 months, *range* = 24–24 months; DCCS age: *M* = 62 months, *SD* = 5 months, *range* = 52–71 months). In terms of the highest level of maternal education within each dyad, three attended high schools, five attended five-year colleges, 25 attended universities, and six attended graduate schools. Raw expressive vocabulary scores of children at 24 months old ranged between 6 and 491 (*M* = 262.1, *SD* = 131.3). All children were typically developing Chinese-Mandarin native speakers, and their parents were local Taiwanese. This study was approved by the Research Ethics Committee of National Taiwan Normal University (REC Number: 201708HM004, 202301HM006). Written informed parental consent was obtained prior to participation.

### Procedures

At 24 months of age, parent-child dyads received a home visit. During this visit, parents were invited to fill in the MacArthur Communicative Development Inventory Taiwan (MCDI-T; 42), and to read with their child using a picture book for five minutes. The reading session was video-recorded. At 5 years of age, children were invited to participate in an fNIRS study in which their frontal activations during the EF task (i.e., DCCS task) were measured.

### Measures

#### DR.

The Chinese version of the picture book “Dear Zoo”, authored by Rod Campbell, was used during the 5-minute shared reading. This is a 16-page book with a different animal hidden behind a small flap on every page. We adopted the DR coding scheme designed by Chang, et al. [41], where the shared reading sessions were videotaped and coded for any behaviour that fits the features of PEER (i.e., prompt, evaluate, expand, and repeat). The ‘prompt’ category was indexed by CROWD (i.e., completion, recall, open-ended, WH questions, and distancing). Table 1 shows the coding scheme. Any of such actions was given a score of one, while utterances that did not fit these DR coding categories were not counted toward the DR score. These scores were summed up to create a DR score for each dyad, where a higher score indicates more parent-child dialogic reading. We used the frequency of parents’ DR strategies as our primary index because our goal was to capture the frequency of DR strategies during shared book reading, rather than the density of DR strategies in parent’s overall speech.

**Table 1:** Coding scheme for the shared reading task.

Strategies	Definitions	Examples of 2-year-olds
**Prompt (P)**	Inviting children to express themselves about the book’s content by using the CROWD strategies.	–
**Completion (C)**	Leaving a sentence hanging for children to complete.	Parent: “The monkey is eating a ___.”
**Recall (R)**	Asking children about an information that was previously read.	Parent: “Which animal was too big and was sent back to the zoo?”
**Open-ended (O)**	Asking children questions that encourage them to respond to the book in their own way.	Parent: “Guess which animal the zoo will send next?”
**WH-questions (W)**	Prompting children using WH question words.	Parent: “What is inside the basket?”
**Distancing (D)**	Asking children to relate the book’s content with their own experience.	Parent: “Did we see elephants the last time we went to the zoo?”
**Evaluate (E)**	Praising or correcting children’s response.	Child: “Monkey” Parent: “Yes, it is a monkey.”
**Expand (E)**	Providing more details to children’s responses.	Child: “monkey…” Parent: “Yes, the monkey is eating a banana.”
**Repeat (R)**	Reinforcing children’s learning by asking them to repeat the correct answer.	Parent: “Say lion.”

*Note.* Adapted from Chang, Hsieh [41]. Every sentence can only be coded for a single PEER strategy. However, for the strategy prompt, multiple CROWD strategies can be coded within a sentence. A sentence in the present study is defined as the shortest possible utterance that expresses some meaning.

In order to code for the PEER features, all dialogues between the parent and the participating child in the video recordings were first transcribed. Two research assistants were trained to code the video recordings. During the training, PI (SW) defined and played video examples of the PEER categories. After the training, both coders coded 13 videos independently, which were used to evaluate inter-coder reliability using the Intraclass Correlation Coefficient (ICC) based on a two-way random effects model [ICC [2, k]; 43]. The estimated ICC for overall PEER score was  .98, 95% CI  [.94,  .99] (Prompt:  .99, 95% CI  [.99,  .99]; Evaluate:  .99, 95% CI  [.97,  .99]; Expand:  .64, 95% CI  [.17,  .87]; Repeat:  .96, 95% CI  [.87,  .98]), indicating effective training and reliable coding. The rest of the videos were then coded by the two coders independently, with each coder coding 13 videos.

#### Neural correlates of EF.

**Fig 1:**
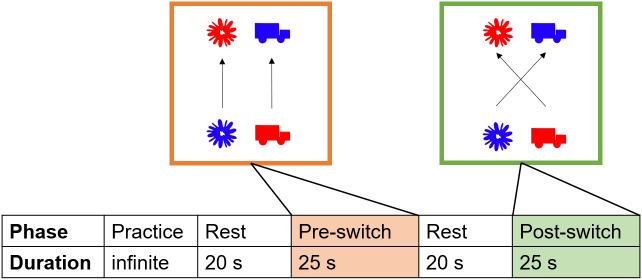
Design of the DCCS task.

Frontal activations associated with EF were measured using fNIRS while children performed the Dimensional Change Card Sort (DCCS) task. The DCCS was carried out as a live demonstration instead of on a computer and had three sessions, where each session presented children with different sets of cards (see S1 Table for the complete stimuli set). In each session, children saw two target cards (e.g., a red flower and a blue truck) pinned on a board and were presented with test cards that matched the target cards on one dimension, i.e., either on colour or on shape (e.g., a blue flower or a red truck). Each session started with a practice phase followed by a rest phase (20 s), a pre-switch phase (5 s of instructions and 20 s of card sorting), a rest phase (20 s), a post-switch phase (5 s of instructions and 20 s of card sorting; see Fig 1), a rest phase (20 s) and finally a mix phase (10 s of instructions and 20 s of card sorting). The mix phase was not presented here as it is not the focus of the current study. For the purpose of the current study, data from the post-switch phase was reported to identify neural correlates of EF in the frontal lobe.

In the pre-switch and post-switch phases, the first five seconds were used to remind children of the sorting rule. Hence, the actual task duration in which children were given test cards to sort was 20 s. In the practice phase, children were allowed up to six practice trials, during which if they failed to sort cards correctly for two consecutive trials, the experiment was stopped.

Children were asked to sort cards by one dimension (e.g., shape) in the pre-switch phase and then by the other dimension (e.g., colour) in the post-switch phase. During the five seconds of instructions in the pre-switch and post-switch phases, children were told the following (when they had to sort cards by the shape dimension): “Now, we are playing the shape game. In the shape game, all the flowers go here [experimenter points at the flower target card], and all the trucks go here [experimenter points at the truck target card]”. When children had to sort cards by the colour dimension, they were told the following: “Now, we are playing the colour game. In the colour game, all the red cards go here [experimenter points at the red target card], and all the blue cards go here [experimenter points at the blue target card]”. The order of the sorting dimensions was the same for all children. The 20-second card-sorting duration allowed children to sort about six test cards. The task followed the pace of each child, which was under the control of the experimenter when providing the test cards. Every time the experimenter handed a test card to the child, the experimenter would say the following: “This is X, where does it go?”. In the rest phase, children were asked to sit quietly while doing nothing. The percentage of correct responses was analysed offline with video recordings.

**Fig 2:**
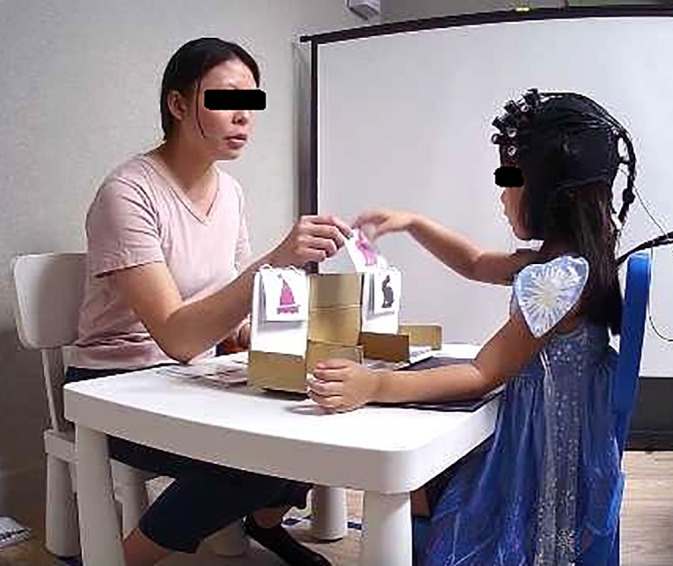
Setup of the fNIRS task.

Before the experiment, the participating child was invited to sit on a child-sized chair. The circumference of the children’s heads was measured (*M* = 50.47 cm; *SD* = 1.13; range = 48–53 cm), and the best-fitting cap was determined. The sizes of easycap used in the current study were 48, 50 and 52 cm. To position the cap accurately, we used a flexible tape to measure the distance from the Nasion to the Inion centrally over the head and the midpoint between the bilateral preauricular points of each child. Then, we placed the cap and positioned the Cz in the intersection of these two midpoints. During the experiment, the experimenter sat opposite to the child (see Fig 2). The children were required to use their right hand to hold the cards while their left hands were placed on the table. The children were also instructed to remain silent during the testing phases of DCCS. The DCCS task lasted about 10 minutes. The DCCS performance of children was video recorded to allow for offline coding of accuracy, which was then used as an exclusion criterion prior to fNIRS data processing.

**Fig 3:**
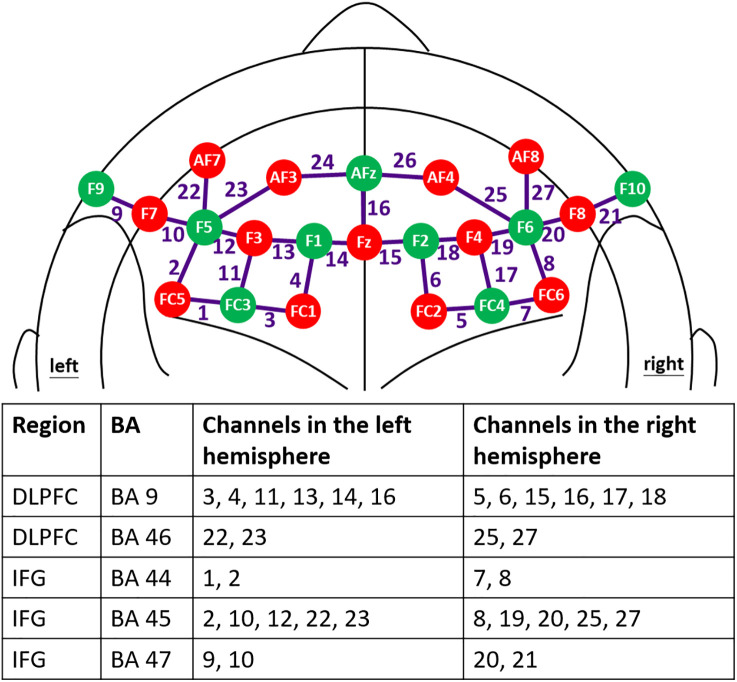
Localisation of channels and brain regions. The green circles refer to detectors while the red circles refer to sources. The numbers beside the purple lines are the channel numbers. The corresponding Brodmann Area (BA) and brain region of these channels are given in the table. DLPFC = dorsolateral prefrontal cortex; IFG = inferior prefrontal gyrus.

The fNIRS recordings were conducted with the NIRSport2 (NIRx Medizintechnik GmbH, Berlin, Germany) CW-NIRS device with 27 channels (13 sources, λ 1∣2 = 760∣850 nm and 9 detectors) placed bilaterally over the PFC regions according to the international 10–20 EEG placement system (see Fig 3). The Optodes Location Decider (fOLD) [44] was used for the initial placement of optodes according to the desired cortical region-of-interest (ROI), namely IFG and DLPFC. The final optode locations were further refined by referencing previous literature. The distance between the source and detector optodes was between 2.5 and 3 cm. The fNIRS data were collected at a sampling rate of 6.26 Hz and converted to concentration changes using the modified Beer-Lambert law (mBLL).

The DCCS accuracy scores were determined offline from the recorded videos. Any experimental block with an accuracy score of less than 80% was treated as an invalid block and discarded. Using this criterion, three children with two or three invalid blocks were excluded from subsequent analyses as they did not have enough blocks for analyses (also see *Participants*). Among the remaining 39 children who comprised the final sample, five of them had one invalid block (five invalid blocks in total). Data from these five blocks were also removed prior to further data processing. In the end, the average accuracy score of the 39 children was 99% (*SD* = 2, *range* = 94–100) during the post-switch phase.

**Fig 4:**
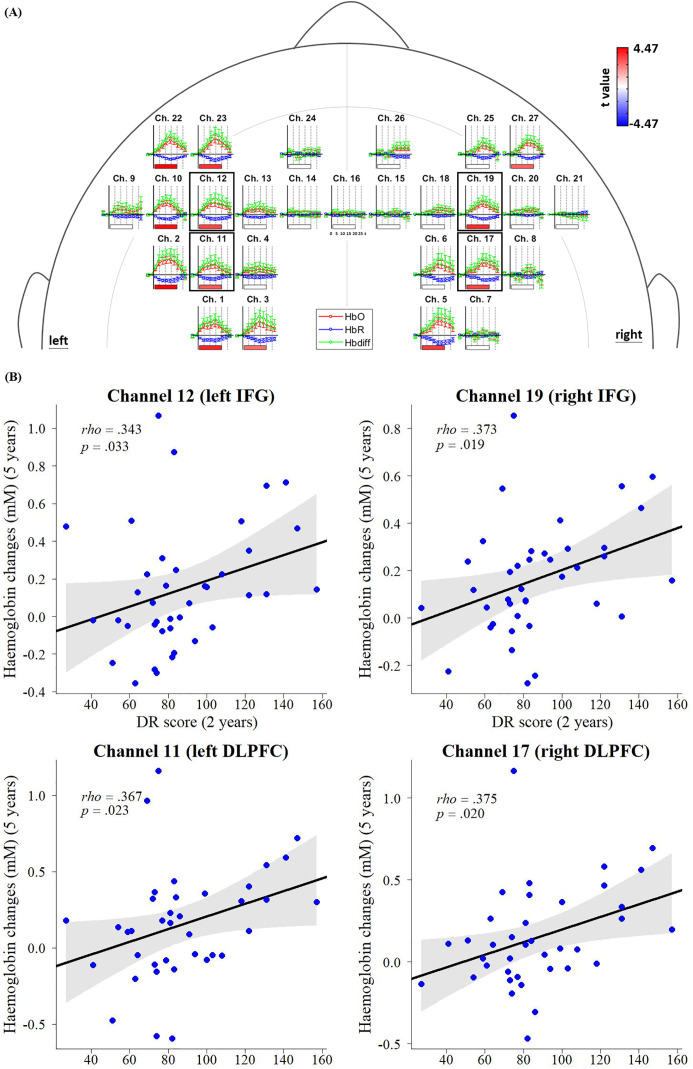
Brain activation map of all channels in the post-switch phase. **(A)** Ch: channel; HbO: oxygenated haemoglobin; HbR: deoxygenated haemoglobin; Hbdiff: the difference between HbO and HbR. Plots with a red horizontal bar refers to channels which were activated significantly in the post-switch phase while plots with a white horizontal bar refers to channels which were not activated significantly. The four black borders indicate channels that correlated significantly with DR score. **(B)** DLPFC: dorsolateral prefrontal cortex; DR: dialogic reading; IFG: inferior frontal gyrus. These scatterplots show the correlation between DR scores and the activation of channels 11, 12, 17, and 19. The black solid lines show the fitted linear regression line while the shaded grey areas represent the 95% confidence interval.

Data from the five seconds of instructions prior to the actual card sorting was not included, therefore only the last 20 seconds (5–25 s) during the post-switch phases were analysed. This time window is also employed in past studies (e.g., 21). The raw fNIRS data were preprocessed using the Homer 3 v1.80.2 package [45] in MATLAB version R2017b [46]. First, bad channels (via visual inspections) and channels showing very high or low optical readings were excluded from further analyses (using the function *hmrR_PruneChannels*, SD range between 25 and 45). The raw intensity data were then converted to optical density (OD) changes. A motion detection filter was then applied to identify motion artefacts (using the function *hmrR_MotionArtifactByChannel*: *tMotion* = 0.5, *tMask* = 1.0, *STDEVthresh* = 5, *AMPthresh* = 0.05). After motion detection, the combination of Spline (*p* = .99) and Wavelet (performed as a second step; *iqr* = 1.50), an effective way of correcting motion artefacts recommended by Di Lorenzo, Pirazzoli [47], was applied for motion corrections. Finally, data were filtered using a band-pass filter between 0.01 Hz and 0.1 Hz to reduce slow drifts and high-frequency noise. The OD data were then converted to concentration changes using the modified Beer-Lambert law [48]. The correlation-based signal improvement was used to improve the signal quality (using the function *hmrR_MotionCorrectCbsi: turnon* = 1*)* [49]. Finally, we calculated a block average for the last 20 seconds (5–25 s) of the post-switch phase. Time courses for the post-switch phase averaged across sessions and participants are shown in Fig 4(A). The subsequent statistical analyses were performed on the block average data of the difference between HbO and HbR (HbDiff).

#### Covariates.

To control for children’s language ability, we measured expressive vocabulary size because Kuhn et al. [29] and Daneri et al. [50] revealed that vocabulary size at a younger age (2–3 years) is predictive of children’s EF at preschool ages (4–5 years). To do so, we used MCDI-T [42]. There are a total of 354 words in the MCDI-T and parents were asked to put a tick mark beside words which their child could produce. A child’s vocabulary score is then calculated by adding all the words which the parents indicated that the child is able to say.

Additionally, considering that socioeconomic status (SES) has been widely reported to be influential on children’s EF [51], we measured maternal education to control for SES. Maternal education was used as a proxy for SES as maternal education has been consistently shown to be strongly associated with children’s cognitive and brain development [27,52,53] among other SES indexes [54]. This information was obtained by asking parents to fill in a background questionnaire. Parents were required to state whether the highest level of education achieved by the mother was middle school or below, high school, vocational school, university, or graduate school.

### Analysis plan

All analyses were computed in R [55] and MATLAB. To identify channels showing significant activations during the DCCS task, one-sample t-tests of the HbDiff changes were performed against a baseline of zero for each channel with FDR correction to avoid false positives. We then examined the Spearman correlation between DR scores and individual channels exhibiting activation significantly above zero baseline after controlling for maternal education and expressive vocabulary of children with FDR correction. In our pre-registered analysis (https://osf.io/hdumq), we planned to group the 27 channels into three clusters, i.e., BA9, BA46, and BA47. However, upon further reflection, we decided that the location of each channel is more definitive and informative than the location of the grouped channels into clusters.

## Results

**Fig 5:**
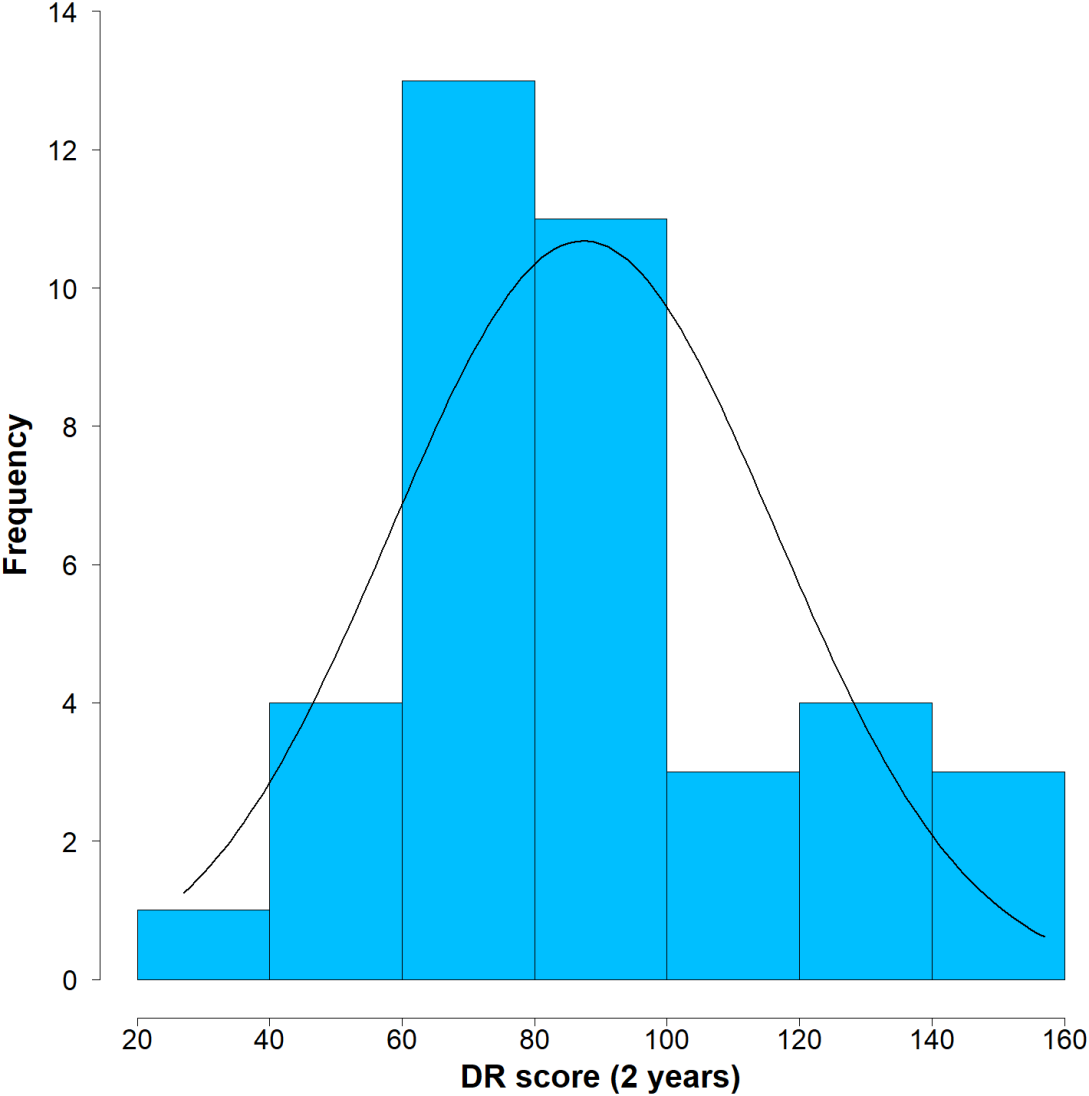
Distribution of DR scores.

Shared reading at 24 months was generally interactive and parents made use of PEER during reading (*M* = 87.36, *SD* = 29.14, range = 27–157). As illustrated in Fig 5, the DR score is normally distributed (Shapiro-Wilk test: *W* = .961, *p* = .212), indicating sufficient individual differences among dyads.

Fig 4(A) displays the mean responses in HbO, HbR, and HbDiff signals during the DCCS post-switch phase. The typical pattern of haemodynamic responses with an increase in HbO and a decrease in HbR was observed. Some channels had slightly less data because we had to remove the channel’s data of some children. Specifically, channels 4 and 6 lost 4 data each while channels 3, 5, 11, 17, 23 lost 1 data each. One-sample t-tests with FDR correction showed that 12 channels [1–3,5,10–12,17,19,22,23,27] exhibited significant changes in HbDiff during the post-switch phase. These channels correspond to left BA44 (channels 1, 2), bilateral BA9 (channels 3, 5, 11, 17), bilateral BA45 (channels 12, 19, 22, 23, 27), bilateral BA46 (channels 22, 23, 27) and left BA47 (channel 10). Among these channels, HbDiff concentration changes of channels 11, 12, 17, and 19 correlated significantly with DR scores (*rho*_*11*_ = .367, *p* = .023; *rho*_*12*_ = .343, *p* = .033; *rho*_*17*_ = .375, *p* = .020; *rho*_*19*_ = .373, *p* = .019) (Table 2; Fig 4b). The correlations remained significant (*rho*_*11*_ = .373, *p* = .036; *rho*_*12*_ = .385, *p* = .030; *rho*_*17*_ = .462, *p* = .007; *rho*_*19*_ = .424, *p* = .016) after maternal education and expressive vocabulary of children were taken into account. The correlation results obtained using HbO and HbR concentration changes showed similar patterns (S2 Table).

**Table 2:** Test statistics for the channels showing significant activation during DCCS and their correlation with DR score.

Ch	Mean (SD)	95% CI	t-statistics	Full correlation	Partial correlation
**1**	0.19 (0.35)	[0.08, 0.30]	t(38)=3.40, *p*=.006, *d*=0.54**	*r*_*s*_=.143, *p*=.385, *q*=.403	*r*_*s*_=.147, *p*=.421
**2**	0.22 (0.35)	[0.10, 0.33]	t(38)=3.87, *p*=.002, *d*=0.62**	*r*_*s*_=.152, *p*=.356, *q*=.381	*r*_*s*_=.079, *p*=.669
**3**	0.20 (0.45)	[0.05, 0.35]	t(38)=2.75, *p*=.021, *d*=0.45*	*r*_*s*_=-.036, *p*=.830, *q*=.830	*r*_*s*_=-.024, *p*=.895
**5**	0.19 (0.39)	[0.06, 0.32]	t(38) =2.98, *p*=.014, *d*=0.48*	*r*_*s*_=.243, *p*=.142, *q*=.170	*r*_*s*_=.301, *p*=.094
**10**	0.16 (0.26)	[0.07, 0.24]	t(38)=3.78, *p*=.002, *d*=0.61**	*r*_*s*_=.068, *p*=.681, *q*=.689	*r*_*s*_=.044, *p*=.810
**11**	0.16 (0.37)	[0.04, 0.28]	t(38)=2.64, *p*=.025, *d*=0.43*	*r*_*s*_=.367, *p*=.023, *q*=.037*	*r*_*s*_=.373, *p*=.036*
**12**	0.15 (0.33)	[0.04, 0.25]	t(38)=2.80, *p*=.020, *d*=0.45*	*r*_*s*_=.343, *p*=.033, *q*=.049*	*r*_*s*_=.385, *p*=.030*
**17**	0.15 (0.31)	[0.05, 0-25]	t(38)=3.02, *p*=.014, *d*=0.49*	*r*_*s*_=.375, *p*=.020, *q*=.034*	*r*_*s*_=.462, *p*=.007**
**19**	0.17 (0.24)	[0.09, 0.24]	t(38)=4.40, *p*=.002, *d*=0.70**	*r*_*s*_=.373, *p*=.019, *q*=.033*	*r*_*s*_=.424, *p*=.016*
**22**	0.16 (0.26)	[0.08, 0.25]	t(38)=3.89, *p*=.002, *d*=0.62**	*r*_*s*_=.197, *p*=.228, *q*=.256	*r*_*s*_=.232, *p*=.202
**23**	0.20 (0.32)	[0.09, 0.30]	t(38)=3.82, *p*=.002, *d*=0.62**	*r*_*s*_=.179, *p*=.284, *q*=.308	*r*_*s*_=.278, *p*=.123
**27**	0.16 (0.25)	[0.07, 0.24]	t(38)=3.28, *p*=.002, *d*=0.61**	*r*_*s*_=.224, *p*=.171, *q*=.199	*r*_*s*_=.170, *p*=.351

Ch: Channel; CI: confidence interval. The one-sample t-tests are conducted on Hbdiff (difference between HbO and HbR). Full and partial correlations are run using Spearman correlation. The *q* values are *p* values that are adjusted based on FDR corrections.

## Discussion

The present study explored the relationship between children’s DR experience at 2 years old and their frontal neural responses associated with EF at 5 years old. Our findings demonstrate a significant positive correlation between the amount of dialogic interaction provided by parents during shared reading at age 2 and the strength of neural activation in key brain regions associated with EF – specifically the bilateral DLPFC (BA9, channels 11 & 17) and bilateral IFG (BA45, channels 12 & 19) – during a DCCS task at age 5. This correlation remained significant even after controlling for maternal education and children’s expressive vocabulary at age 2, suggesting a robust relationship between early DR experience and later neural correlates of EF. These results imply that early DR experience may cultivate the neural infrastructure necessary for EF development.

As outlined in the *Introduction*, DR is a form of shared reading in which parents employ PEER strategies while reading with their children. This approach assists children in utilising their EF to process information associated with the book that they are reading, to regulate their interaction with their parents, and so on. This heightened EF involvement provides children with continuous opportunities to facilitate their frontal lobe development. As a consequence, DR not only enables parents to scaffold children’s learning but also contributes to shaping children’s developing brain circuitry in the frontal area associated with EF. The current findings support this notion.

The current results also align with the prior literature that highlights the neurobiological benefits of interactive shared reading experiences [4,8,26,56]. However, our study expands upon previous work in several crucial ways. First, it addresses limitations of prior research by demonstrating the longitudinal effects of DR at a young age (2 years old) on prefrontal neural activity during an explicit and classic EF task (DCCS) at a later age (5 years old). The persistence of these effects three years later suggest that DR introduced at age 2 may contribute to long-term neural organization supporting EF. The frontal cortex undergoes rapid synaptic growth during the first 2 years after birth [57], followed by extensive pruning and myelination that continue through early childhood but slow down thereafter [58]. This early period of neural change represents a window of heightened plasticity during which experience-dependent activities, such as DR, may exert especially strong and lasting effects on the developing executive network. Engaging toddlers in DR at age 2 may thus lay a durable neural foundation that supports later self-regulation and goal-directed behaviour.

Second, the use of fNIRS in the current study allows for more precise localization of brain activity compared to EEG, providing additional understanding of how DR relates to functions of specific brain regions involved in EF. Also, by quantifying DR through PEER coding scheme [41], the level of parent-child dialogic interaction during shared reading was directly measured, offering a more nuanced understanding of how the intensity of DR interactions contributes to brain development.

The findings that increased DR interaction from parents at age 2 is linked to stronger DLPFC and IFG activation at age 5 during an EF task is particularly significant. The DLPFC is crucial for higher-order cognitive functions such as working memory [59] and cognitive shifting [21], while the IFG plays a vital role in inhibitory control and selective attention [60] All are integral components of EF. Therefore, our results provide compelling and supplementary evidence that DR practices can effectively strengthen the neural pathways underpinning EF.

Notably, we observed individual variability in the strength of activation in the DLPFC and IFG, despite similar behavioural performance among children. This implies that brain activity offers a more nuanced understanding of cognitive processes compared to behavioural measures alone. Identical behavioural outcomes may arise from varying cognitive processing quality, and brain data is more sensitive in detecting these subtle differences. Such differences may stem from factors other than children’s DR experience, including genetic predispositions [23], SES [27], children’s language ability [29] and other environmental factors. To mitigate the impact of these confounding factors, we controlled for maternal education (a robust proxy of SES, 27) and children’s expressive vocabulary and found the link between DR experiences and neural activation related to EF persists.

The persistent positive correlation between the strength of neural activation and the richness of DR experiences thus reinforces the idea that intense DR interactions can facilitate the development of neural pathways related to EF. Such findings lend support to the notion that interactive reading not only enriches children’s language development but also lays the groundwork for critical cognitive skills necessary for future academic success. This conclusion is further strengthened by the fact that the DCCS task used in the current study requires minimal language processing compared to the story listening task widely used in prior research (e.g., 4).

The current findings have considerable practical implications on parenting practices, particularly regarding the role of shared reading as a customizable aspect of the home environment that can significantly influence children’s brain development. The significant positive correlation found between the extent of parents’ dialogic interactions during shared reading at age 2 and enhanced neural activation in brain regions associated with EF at age 5 suggests that DR can effectively cultivate the foundational neural architecture necessary for cognitive development. Studies have shown that even with non-verbal infants, parent-child shared reading stimulates more adult-child language interaction compared to other daily activities, such as toy play [61,62], singing songs, mealtime, and personal care [62]. This emphasizes the unique role of shared reading in providing opportunities for serve-and-return interactions. Importantly, prior research and our current data have both shown substantial individual differences in the amount of dialogic strategies used during shared reading among parents [41]. This suggests the need for educational programs to provide training and resources that assists parents to implement effective reading practices.

Despite the significant findings of the current study, there are several notable limitations. First, our final sample size is only 39 parent-child dyads, which is relatively small. Thus, caution is needed when interpreting our findings. The study design is also correlational, which limits the ability to establish cause-and-effect relationships between early DR and later neural development associated with EF. Notably, we lack data on DR experiences for children at age 5, preventing us from ruling out the influence of their current DR experiences on our findings and asserting that DR experiences at age 2 directly impact the strength of brain activation associated with EF at age 5. Additional studies are necessary and warranted to address this gap and provide further insight into this issue. Moreover, other underlying variables, such as genetic predispositions may also play a role in shaping both parenting practices and children’s neurodevelopment, despite our efforts to control for maternal education as an SES proxy and children’s expressive vocabulary. Future studies should include and control for other influential factors. Furthermore, while the study quantitatively assessed the level of parent-child dialogic interaction using the PEER coding scheme, it may not fully capture the complexity of parent-child dynamics during shared reading, such as non-verbal cues and other forms of engagement that could also influence outcomes. Future research should aim to provide a more comprehensive understanding of the complexities of DR and its various effects on children’s brain development.

## Conclusions

To sum up, our study demonstrates a robust link between interactive DR engagement at age 2 and the strength of neural activation in the bilateral DLPFC (BA9) and IFG (BA45) – the key brain regions associated with EF – at age 5. These findings emphasize the importance of promoting interactive shared reading in early childhood to support the neural development associated with crucial cognitive skills essential for academic success. The results strongly advocate for the inclusion of effective DR interventions in early childhood education programs.

## Supporting information

S1 Table Full stimuli set of the DCCS task. (DOCX)

S2 Table Spearman’s correlation between SBR and the significant channels for oxygenated haemoglobin (HbO) and deoxygenated haemoglobin (HbR), with their respective *p* values and *q* values (i.e., *p* values that are adjusted based on FDR corrections). (DOCX)

## References

[1] Mol SE, Bus AG, de Jong MT, Smeets DJH. Added Value of Dialogic Parent–Child Book Readings: A Meta-Analysis. Early Education and Development. 2008;19(1):7–26. doi: 10.1080/10409280701838603

[2] Pecukonis M, Yücel M, Lee H, Knox C, Boas DA, Tager-Flusberg H. Do Children’s Brains Function Differently During Book Reading and Screen Time? A fNIRS Study. Dev Sci. 2025;28(2):e13615. doi: 10.1111/desc.13615 39888180

[3] Hutton JS, Horowitz-Kraus T, Mendelsohn AL, DeWitt T, Holland SK, C-MIND Authorship Consortium. Home Reading Environment and Brain Activation in Preschool Children Listening to Stories. Pediatrics. 2015;136(3):466–78. doi: 10.1542/peds.2015-0359 26260716 PMC9923605

[4] Hutton JS, Phelan K, Horowitz-Kraus T, Dudley J, Altaye M, DeWitt T, et al. Shared Reading Quality and Brain Activation during Story Listening in Preschool-Age Children. J Pediatr. 2017;191:204–211.e1. doi: 10.1016/j.jpeds.2017.08.037 29173308 PMC5728185

[5] Farah R, Meri R, Kadis DS, Hutton J, DeWitt T, Horowitz-Kraus T. Hyperconnectivity during screen-based stories listening is associated with lower narrative comprehension in preschool children exposed to screens vs dialogic reading: An EEG study. PLoS One. 2019;14(11):e0225445. doi: 10.1371/journal.pone.0225445 31756207 PMC6874384

[6] Zhai Y, Xie H, Zhao H, Wang W, Lu C. Neural synchrony underlies the positive effect of shared reading on children’s language ability. Cereb Cortex. 2023;33(19):10426–40. doi: 10.1093/cercor/bhad293 37562850

[7] Zivan M, Gashri C, Habuba N, Horowitz-Kraus T. Reduced mother-child brain-to-brain synchrony during joint storytelling interaction interrupted by a media usage. Child Neuropsychol. 2022;28(7):918–37. doi: 10.1080/09297049.2022.2034774 35129078

[8] Twait E, Farah R, Shamir N, Horowitz-Kraus T. Dialogic reading vs screen exposure intervention is related to increased cognitive control in preschool-age children. Acta Paediatr. 2019;108(11):1993–2000. doi: 10.1111/apa.14841 31074876

[9] Wang S, Tzeng OJL, Aslin RN. Predictive brain signals mediate association between shared reading and expressive vocabulary in infants. PLoS One. 2022;17(8):e0272438. doi: 10.1371/journal.pone.0272438 35921370 PMC9348734

[10] Diamond A. Executive functions. Annu Rev Psychol. 2013;64:135–68. doi: 10.1146/annurev-psych-113011-143750 23020641 PMC4084861

[11] Whitehurst GJ, Falco FL, Lonigan CJ, Fischel JE, DeBaryshe BD, Valdez-Menchaca MC, et al. Accelerating language development through picture book reading. Developmental Psychology. 1988;24(4):552–9. doi: 10.1037/0012-1649.24.4.552

[12] Zevenbergen AA, Whitehurst GJ. Dialogic reading: A shared picture book reading intervention for preschoolers. In: van Kleeck A, Stahl SA, Bauer EB,s editors. On reading books to children: Parents and teachers. Mahwah, NJ: Lawrence Erlbaum Associates Publishers. 2003:170–200.

[13] Baddeley A. Exploring the Central Executive. The Quarterly Journal of Experimental Psychology Section A. 1996;49(1):5–28. doi: 10.1080/713755608

[14] Baddeley AD, Hitch G. Working Memory. Psychology of Learning and Motivation. Elsevier. 1974:47–89. doi: 10.1016/s0079-7421(08)60452-1

[15] Friedman NP, Miyake A. The relations among inhibition and interference control functions: a latent-variable analysis. J Exp Psychol Gen. 2004;133(1):101–35. doi: 10.1037/0096-3445.133.1.101 14979754

[16] Zelazo PD, Müller U, Frye D, Marcovitch S, Argitis G, Boseovski J, et al. The development of executive function in early childhood. Monogr Soc Res Child Dev. 2003;68(3):vii–137. doi: 10.1111/j.0037-976x.2003.00260.x 14723273

[17] Buss AT, Fox N, Boas DA, Spencer JP. Probing the early development of visual working memory capacity with functional near-infrared spectroscopy. Neuroimage. 2014;85 Pt 1(0 1):314–25. doi: 10.1016/j.neuroimage.2013.05.034 23707803 PMC3859697

[18] Gathercole SE, Pickering SJ, Ambridge B, Wearing H. The structure of working memory from 4 to 15 years of age. Dev Psychol. 2004;40(2):177–90. doi: 10.1037/0012-1649.40.2.177 14979759

[19] Diamond A, Kirkham N, Amso D. Conditions under which young children can hold two rules in mind and inhibit a prepotent response. Dev Psychol. 2002;38(3):352–62. doi: 10.1037//0012-1649.38.3.352 12005379

[20] Simpson A, Riggs KJ. Factors responsible for performance on the day-night task: response set or semantics? Developmental Science. 2005;8(4):360–71. 15985070 10.1111/j.1467-7687.2005.00424.x

[21] Moriguchi Y, Hiraki K. Neural origin of cognitive shifting in young children. Proc Natl Acad Sci U S A. 2009;106(14):6017–21. doi: 10.1073/pnas.0809747106 19332783 PMC2667026

[22] Kanemura H, Aihara M, Aoki S, Araki T, Nakazawa S. Development of the prefrontal lobe in infants and children: a three-dimensional magnetic resonance volumetric study. Brain Dev. 2003;25(3):195–9. doi: 10.1016/s0387-7604(02)00214-0 12689699

[23] Huttenlocher PR, Dabholkar AS. Regional differences in synaptogenesis in human cerebral cortex. J Comp Neurol. 1997;387(2):167–78. doi: 10.1002/(sici)1096-9861(19971020)387:2<167::aid-cne1>3.0.co;2-z 9336221

[24] Deoni SCL, Mercure E, Blasi A, Gasston D, Thomson A, Johnson M, et al. Mapping infant brain myelination with magnetic resonance imaging. J Neurosci. 2011;31(2):784–91. doi: 10.1523/JNEUROSCI.2106-10.2011 21228187 PMC6623428

[25] Chugani HT, Phelps ME. Maturational changes in cerebral function in infants determined by 18FDG positron emission tomography. Science. 1986;231(4740):840–3. doi: 10.1126/science.3945811 3945811

[26] Ohgi S, Loo KK, Mizuike C. Frontal brain activation in young children during picture book reading with their mothers. Acta Paediatr. 2010;99(2):225–9. doi: 10.1111/j.1651-2227.2009.01562.x 19849672 PMC2848291

[27] Zhu C, Chen Y, Müller H-G, Wang J-L, O’Muircheartaigh J, Bruchhage M, et al. Trajectories of brain volumes in young children are associated with maternal education. Hum Brain Mapp. 2023;44(8):3168–79. doi: 10.1002/hbm.26271 36896867 PMC10171562

[28] Dai X, Hadjipantelis P, Wang J-L, Deoni SCL, Müller H-G. Longitudinal associations between white matter maturation and cognitive development across early childhood. Hum Brain Mapp. 2019;40(14):4130–45. doi: 10.1002/hbm.24690 31187920 PMC6771612

[29] Kuhn LJ, Willoughby MT, Vernon-Feagans L, Blair CB, Family Life Project Key Investigators. The contribution of children’s time-specific and longitudinal expressive language skills on developmental trajectories of executive function. J Exp Child Psychol. 2016;148:20–34. doi: 10.1016/j.jecp.2016.03.008 27101154 PMC9154006

[30] Leech KA, McNally S, Daly M, Corriveau KH. Unique effects of book-reading at 9-months on vocabulary development at 36-months: Insights from a nationally representative sample of Irish families. Early Childhood Research Quarterly. 2022;58:242–53. doi: 10.1016/j.ecresq.2021.09.009

[31] Niklas F, Cohrssen C, Tayler C. The sooner, the better: Early reading to children. Sage Open. 2016;6(4):2158244016672715.

[32] Jimenez ME, Reichman NE, Mitchell C, Schneper L, McLanahan S, Notterman DA. Shared reading at age 1 year and later vocabulary: A gene-environment study. The Journal of Paediatrics. 2020;216:189–96. 10.1016/j.jpeds.2019.07.008 PMC6917887 31402141

[33] Zelazo PD. The Dimensional Change Card Sort (DCCS): a method of assessing executive function in children. Nat Protoc. 2006;1(1):297–301. doi: 10.1038/nprot.2006.46 17406248

[34] Milner B. Some effects of frontal lobectomy in man. In: Warren J, Akert K, editors. The Frontal Granular Cortex and Behavior. New York: McGraw-Hill. 1964:313–34.

[35] Moriguchi Y, Hiraki K. Prefrontal cortex and executive function in young children: a review of NIRS studies. Front Hum Neurosci. 2013;7:867. doi: 10.3389/fnhum.2013.00867 24381551 PMC3865781

[36] Wang J, Sakata C, Moriguchi Y. The neurobehavioral relationship between executive function and creativity during early childhood. Dev Psychobiol. 2021;63(7):e22191. doi: 10.1002/dev.22191 34674250

[37] Li H, Wu D, Yang J, Xie S, Luo J, Chang C. A Functional Near-Infrared Spectroscopy Examination of the Neural Correlates of Cognitive Shifting in Dimensional Change Card Sort Task. Front Hum Neurosci. 2021;14:561223. doi: 10.3389/fnhum.2020.561223 33551771 PMC7859114

[38] Li H, Wu D, Yang J, Xie S, Chang C, Luo J. Bilinguals have more effective executive function: Evidence from an fNIRS study of the neural correlates of cognitive shifting. International Journal of Bilingualism. 2022;27(1):22–38. doi: 10.1177/13670069221076375

[39] Moriguchi Y. Relationship between cool and hot executive function in young children: A near-infrared spectroscopy study. Dev Sci. 2022;25(2):e13165. doi: 10.1111/desc.13165 34327776

[40] Moriguchi Y, Hiraki K. Longitudinal development of prefrontal function during early childhood. Dev Cogn Neurosci. 2011;1(2):153–62. doi: 10.1016/j.dcn.2010.12.004 22436437 PMC6987577

[41] Chang CS, Hsieh F-J, Chen T, Wu SC, Tzeng OJL, Wang S. Revisiting Dialogic Reading Strategies with 12-Month-Old Infants. Early Childhood Educ J. 2022;51(8):1413–26. doi: 10.1007/s10643-022-01385-4

[42] Liu HM, Chen YC. Developmental changes in the content and composition of early expressive vocabulary in Mandarin-speaking infants and toddlers. Bulletin of Educational Psychology. 2015;47(2):217–42.

[43] Shrout PE, Fleiss JL. Intraclass correlations: uses in assessing rater reliability. Psychol Bull. 1979;86(2):420–8. doi: 10.1037//0033-2909.86.2.420 18839484

[44] Zimeo Morais GA, Balardin JB, Sato JR. fNIRS Optodes’ Location Decider (fOLD): a toolbox for probe arrangement guided by brain regions-of-interest. Sci Rep. 2018;8(1):3341. doi: 10.1038/s41598-018-21716-z 29463928 PMC5820343

[45] Huppert TJ, Diamond SG, Franceschini MA, Boas DA. HomER: a review of time-series analysis methods for near-infrared spectroscopy of the brain. Appl Opt. 2009;48(10):D280–98. doi: 10.1364/ao.48.00d280 19340120 PMC2761652

[46] MathWorks Inc. MATLAB version: 9.3 (R2017b). Natick, Massachusetts: The MathWorks Inc. 2017.

[47] Di Lorenzo R, Pirazzoli L, Blasi A, Bulgarelli C, Hakuno Y, Minagawa Y, et al. Recommendations for motion correction of infant fNIRS data applicable to multiple data sets and acquisition systems. Neuroimage. 2019;200:511–27. doi: 10.1016/j.neuroimage.2019.06.056 31247300

[48] Cope M, Delpy DT. System for long-term measurement of cerebral blood and tissue oxygenation on newborn infants by near infra-red transillumination. Med Biol Eng Comput. 1988;26(3):289–94. doi: 10.1007/BF02447083 2855531

[49] Cui X, Bray S, Reiss AL. Functional near infrared spectroscopy (NIRS) signal improvement based on negative correlation between oxygenated and deoxygenated hemoglobin dynamics. Neuroimage. 2010;49(4):3039–46. doi: 10.1016/j.neuroimage.2009.11.050 19945536 PMC2818571

[50] Daneri MP, Blair C, Kuhn LJ, FLP Key Investigators. Maternal Language and Child Vocabulary Mediate Relations Between Socioeconomic Status and Executive Function During Early Childhood. Child Dev. 2019;90(6):2001–18. doi: 10.1111/cdev.13065 29707764 PMC6207477

[51] Hackman DA, Gallop R, Evans GW, Farah MJ. Socioeconomic status and executive function: developmental trajectories and mediation. Dev Sci. 2015;18(5):686–702. doi: 10.1111/desc.12246 25659838

[52] Bradley RH, Corwyn RF. Socioeconomic status and child development. Annu Rev Psychol. 2002;53:371–99. doi: 10.1146/annurev.psych.53.100901.135233 11752490

[53] Chen Y, Dubey P, Müller H-G, Bruchhage M, Wang J-L, Deoni S. Modeling sparse longitudinal data in early neurodevelopment. Neuroimage. 2021;237:118079. doi: 10.1016/j.neuroimage.2021.118079 34000395

[54] González L, Cortés-Sancho R, Murcia M, Ballester F, Rebagliato M, Rodríguez-Bernal CL. The role of parental social class, education and unemployment on child cognitive development. Gac Sanit. 2020;34(1):51–60. doi: 10.1016/j.gaceta.2018.07.014 30390996

[55] R Core Team. R: A Language and Environment for Statistical Computing. Vienna, Austria: R Foundation for Statistical Computing. 2023.

[56] Horowitz-Kraus T, Magaliff LS, Schlaggar BL. Neurobiological Evidence for the Benefit of Interactive Parent–Child Storytelling: Supporting Early Reading Exposure Policies. Policy Insights from the Behavioral and Brain Sciences. 2023;11(1):51–8. doi: 10.1177/23727322231217461

[57] Matsuzawa J, Matsui M, Konishi T, Noguchi K, Gur RC, Bilker W, et al. Age-related volumetric changes of brain gray and white matter in healthy infants and children. Cereb Cortex. 2001;11(4):335–42. doi: 10.1093/cercor/11.4.335 11278196

[58] Tanaka C, Matsui M, Uematsu A, Noguchi K, Miyawaki T. Developmental trajectories of the fronto-temporal lobes from infancy to early adulthood in healthy individuals. Dev Neurosci. 2012;34(6):477–87. doi: 10.1159/000345152 23257954

[59] Chai WJ, Abd Hamid AI, Abdullah JM. Working Memory From the Psychological and Neurosciences Perspectives: A Review. Front Psychol. 2018;9:401. doi: 10.3389/fpsyg.2018.00401 29636715 PMC5881171

[60] Aron AR, Robbins TW, Poldrack RA. Inhibition and the right inferior frontal cortex: one decade on. Trends Cogn Sci. 2014;18(4):177–85. doi: 10.1016/j.tics.2013.12.003 24440116

[61] Wu S-C, Tzeng OJL, Wang S. Mothers used wider vocabulary and talked to their six-month-old infants more during shared book reading than when they played with toys. Acta Paediatr. 2024;113(1):84–90. doi: 10.1111/apa.17004 37861073

[62] Clemens LF, Kegel CAT. Unique contribution of shared book reading on adult-child language interaction. J Child Lang. 2021;48(2):373–86. doi: 10.1017/S0305000920000331 32524924

