# Supplementary material for: Dialogic reading at age 2 is linked to frontal activation related to executive function at age 5: An fNIRS study
Source: PLoS One. 2026 Jun 15;21(6):e0351177. doi: 10.1371/journal.pone.0351177 (PMC13268133; doi:10.1371/journal.pone.0351177)
Supplement: S1 Table — (DOCX) [file pone.0351177.s001.docx]

# Supporting information

**S1 Table. Full stimuli set of the DCCS task.**

| Set | Target cards | Test cards | Phase | Order of test trials |
| --- | --- | --- | --- | --- |
| 1 | 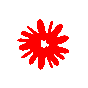 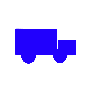 | 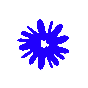 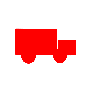 | Pre-switch | flower, truck, flower, truck, flower, truck |
|  |  |  | Post-switch | red, blue, red, blue, blue, red |
| 2 | 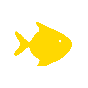 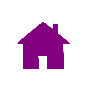 | 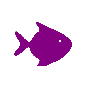 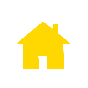 | Pre-switch | yellow, purple, yellow, purple, purple, yellow |
|  |  |  | Post-switch | fish, house, house, fish, house, fish |
| 3 | 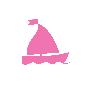 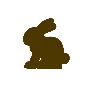 | 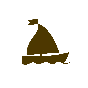 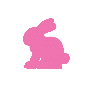 | Pre-switch | pink, brown, pink, brown, brown, pink |
|  |  |  | Post-switch | boat, rabbit, boat, rabbit, boat, rabbit |
