# Supplementary material for: Dialogic reading at age 2 is linked to frontal activation related to executive function at age 5: An fNIRS study
Source: PLoS One. 2026 Jun 15;21(6):e0351177. doi: 10.1371/journal.pone.0351177 (PMC13268133; doi:10.1371/journal.pone.0351177)
Supplement: S2 Table — (DOCX) [file pone.0351177.s002.docx]

# Supporting information

**S2 Table. Spearman’s correlation between SBR and the significant channels for oxygenated haemoglobin (HbO) and deoxygenated haemoglobin (HbR), with their respective *p* values and *q* values (i.e., *p* values that are adjusted based on FDR corrections)**.

| Channels | HbO | | | HbR | | |
| --- | --- | --- | --- | --- | --- | --- |
|  | Mean (SD) | rho | *p* value (*q* value) | Mean (SD) | rho | *p* value (*q* value) |
| 1 | 0.13 (0.24) | .146 | .376 (.398) | -0.06 (0.12) | -.137 | .404 (.427) |
| 2 | 0.16 (0.25) | .132 | .422 (.442) | -0.06 (0.11) | -.167 | .310 (.332) |
| 3 | 0.14 (0.33) | -.010 | .953 (.953) | -0.06 (0.13) | .052 | .756 (.756) |
| 5 | 0.12 (0.27) | .249 | .131 (.155) | -0.07 (0.13) | -.258 | .118 (.151) |
| 10 | 0.11 (0.19) | .037 | .824 (.833) | -0.05 (0.08) | -.103 | .531 (.543) |
| 11 | 0.11 (0.26) | .350 | .031 (.047) | -0.05 (0.11) | -.400 | .013 (.028) |
| 12 | 0.11 (0.24) | .353 | .027 (.041) | -0.04 (0.09) | -.332 | .039 (.059) |
| 13 | 0.05 (0.19) | .236 | .148 (.172) | -0.02 (0.09) | -.215 | .189 (.221) |
| 17 | 0.10 (0.22) | .386 | .017 (.029) | -0.05 (0.09) | -.395 | .014 (.030) |
| 19 | 0.12 (0.17) | .365 | .022 (.037) | -0.05 (0.07) | -.373 | .019 (.036) |
| 22 | 0.12 (0.20) | .182 | .267 (.296) | -0.04 (0.07) | -.252 | .121 (.153) |
| 23 | 0.14 (0.23) | .181 | .276 (.302) | -0.05 (0.09) | -.115 | .490 (.507) |
| 27 | 0.11 (0.19) | .225 | .168 (.194) | -0.04 (0.07) | -.220 | .178 (.213) |
